# Photosynthetic complex stoichiometry dynamics in higher plants: environmental acclimation and photosynthetic flux control

**DOI:** 10.3389/fpls.2014.00188

**Published:** 2014-05-13

**Authors:** Mark A. Schöttler, Szilvia Z. Tóth

**Affiliations:** Max Planck Institute of Molecular Plant PhysiologyPotsdam-Golm, Germany

**Keywords:** acclimation, ATP synthase, cytochrome b_6_f complex, photosystem I, photosystem II, plastocyanin, photosynthetic flux control, senescence

## Abstract

The composition of the photosynthetic apparatus of higher plants is dynamically adjusted to long-term changes in environmental conditions such as growth light intensity and light quality, and to changing metabolic demands for ATP and NADPH imposed by stresses and leaf aging. By changing photosynthetic complex stoichiometry, a long-term imbalance between the photosynthetic production of ATP and NADPH and their metabolic consumption is avoided, and cytotoxic side reactions are minimized. Otherwise, an excess capacity of the light reactions, relative to the demands of primary metabolism, could result in a disturbance of cellular redox homeostasis and an increased production of reactive oxygen species, leading to the destruction of the photosynthetic apparatus and the initiation of cell death programs. In this review, changes of the abundances of the different constituents of the photosynthetic apparatus in response to environmental conditions and during leaf ontogenesis are summarized. The contributions of the different photosynthetic complexes to photosynthetic flux control and the regulation of electron transport are discussed.

## INTRODUCTION

As sessile organisms, higher plants must cope with strong changes in their biotic and abiotic environment. Within seconds to minutes, rapid alterations in light intensity and light quality can occur due to changing cloud cover or due to sun flecks when plants grow in dense canopies. During a clear day, slow ramped changes in light intensity and light quality, but also of temperature and water availability affect photosynthesis. Over weeks to months, photoperiod and nutrient availability may also change. An altered canopy density may result in different levels of shading of leaves, modifying both the light intensity and especially the light quality available for photosynthesis.

Usually, only long-term changes in environmental parameters initiate major adjustments of the composition of the photosynthetic apparatus, a process termed “acclimation” (Walters, 2005). Short-term fluctuations in environmental parameters occurring during the day do not initiate major adjustments of the composition of the photosynthetic apparatus, but instead trigger reversible modifications of photosynthetic light utilization. Changes in *spectral quality* result in imbalanced excitation rates of the two photosystems, especially at low actinic light intensities. Then, “state transitions” are initiated (Allen, 1992; Allen and Forsberg, 2001). An excess excitation of photosystem II (PSII) reduces the plastoquinone pool and activates the thylakoid kinase STN7, which phosphorylates the light harvesting complexes (LHCII; Bellafiore et al., 2005). Upon phosphorylation, peripheral “extra” LHCII trimers detach from the PSII supercomplex and migrate to photosystem I (PSI). Thereby, the PSII antenna cross section is decreased, and the PSI antenna cross section is increased, so that the excitation rates of the two photosystems are rebalanced and the plastoquinone pool becomes oxidized again (Wientjes et al., 2013a). This inactivates STN7 again, and the LHCII will gradually become dephosphorylated by the thylakoid associated phosphatase PPH1 / TAP38 (Pribil et al., 2010; Shapiguzov et al., 2010; Pesaresi et al., 2011). Thus, via reversible redistributions of the “extra” fraction of peripheral LHCII between PSII and PSI, the photosynthetic apparatus can adjust to rapid changes in light quality without any need for adjustments of photosystem stoichiometry.

Also rapid fluctuations in actinic light *intensity* above the level of light saturation of electron transport and assimilation do not trigger stoichiometry adjustments of the photosynthetic complexes. Instead, they activate chloroplast movements as part of a light avoidance response (“photorelocation,” Morita and Nakamura, 2012) and multiple mechanisms resulting in “non-photochemical quenching” (NPQ) of excess excitation energy are activated (recently reviewed by: Horton, 2012; Niyogi and Truong, 2013). Under fluctuating light conditions, especially the photoprotective qE component of NPQ plays a major role. It can be rapidly induced upon high-light exposure, and relaxes within a few minutes after the light intensity drops again below light saturation of photosynthesis. qE is activated by the acidification of the thylakoid lumen, which occurs when the rate of proton influx via linear and possibly cyclic electron flux exceeds the rate of proton efflux through chloroplast ATP synthase (Kanazawa and Kramer, 2002; Avenson et al., 2004). Lumen acidification induces qE via two major mechanisms in higher plants: protonation of two glutamate residues on the luminal site of the PsbS protein (Li et al., 2004), and the de-epoxidation of the xanthophyll violaxanthin via anteraxanthin to zeaxanthin, a reaction catalyzed by the luminal enzyme violaxanthin de-epoxidase (VDE), which is active only at acidic pH values (Arnoux et al., 2009). Together, these processes rapidly increase the rate of thermal dissipation of excess excitation energy in the PSII antenna bed.

Also, supercomplex composition (Minagawa, 2013) and even thylakoid structure can undergo rapid changes in response to light (Kirchhoff et al., 2011; Anderson et al., 2012; Herbstova et al., 2012; Kirchhoff, 2013). None of these changes alter the maximum possible rate of photosynthetic electron transport, i.e., its capacity to produce ATP and NADPH for carbon assimilation and other reactions of primary metabolism. They solely affect the light response and actual quantum yield of photosynthesis.

Only long-term changes in environmental parameters initiate adjustments of photosynthetic complex contents (“acclimation”). Depending on the environmental perturbation and the complex, lag times of many hours to several days have been reported, and the fully acclimated state of the photosynthetic apparatus is only reached after several days to more than one week (Chow and Anderson, 1987; Kim et al., 1993). Different to the rapidly reversible adjustments, which only alter the light response and quantum efficiency of photosynthesis, the long-term acclimation of the photosynthetic apparatus alters photosynthetic capacity. However, the abundances of PsbS and VDE are also adjusted during acclimation, resulting in an altered light response and strength of qE (see below).

Long-term changes in light quality and light intensity directly affect the photosynthetic light reactions. The consequences of changes in plant nutrition, water availability and temperature are often more indirect, mainly via alterations in the activity of the Calvin-Benson cycle and of the subsequent reactions of plant primary metabolism. Low temperature slows down the biochemical reactions of metabolism, while the temperature dependence of photosynthetic electron transport is much lower (Öquist and Huner, 2003). Drought restricts CO_2_ availability to the Calvin-Benson cycle due to stomatal closure, which reduces water loss via transpiration. It also alters the activities of some Calvin-Benson cycle enzymes (Flexas et al., 2004; Lawlor and Tezara, 2009). Consequently, similar to cold stress conditions, Calvin-Benson cycle activity and the metabolic consumption of ATP and NADPH decrease. This has to trigger adjustments of the light reactions, because an imbalance between photosynthetic ATP and NADPH production and their metabolic consumption could disturb the cellular redox homeostasis, and especially a NADP^+^ limitation could cause electron transfer to alternative acceptors such as molecular oxygen. In higher plants, the capacity of the Mehler-Asada cycle to detoxify reactive oxygen species (ROS) generated at the PSI acceptor side may not exceed one to ten percent of the capacity of linear electron flux (Badger et al., 2000; Ruuska et al., 2000; Shirao et al., 2013). Moreover, several stress factors, namely cold stress, may directly limit the activity of ROS scavenging enzymes.

In addition to the Mehler-Asada cycle, at least three other pathways could alleviate a short-term imbalance of electron transport and the metabolic demand, preventing a massive over-reduction of the photosynthetic apparatus and of the chloroplast stroma. The plastid terminal oxidase (PTOX) accepts electrons from plastoquinol and reduces O_2_, releasing water on the stromal side of the membrane and thereby allowing the build-up of a proton motive force across the thylakoid membrane (Lennon et al., 2003). However, in mature leaves of most C_3_ plants, the abundance and activity of PTOX is extremely low (Lennon et al., 2003; Shirao et al., 2013). PTOX mainly regenerates plastoquinone as an electron acceptor for phytoene desaturase during carotenoid biosynthesis during early chloroplast development (Carol et al., 1999; Wu et al., 1999). A high capacity of PTOX in fully developed leaves has so far only been reported for the halophyte *Thellungiella halophila* in response to salt stress (Stepien and Johnson, 2009) and for sun-exposed plants of the high mountain species *Ranunculus glacialis* (Streb et al., 2005; Laureau et al., 2013). Because of the short vegetation period in the alpine mountains, *Ranunculus* maintains a high photosynthetic electron transport capacity even under adverse conditions, i.e., high light intensity combined with low temperature. To avoid an over-reduction of the electron transport chain at low temperatures, *Ranunculus* accumulates at least 100-fold higher levels of PTOX than normal C_3 _plants (Streb et al., 2005).

Besides of the Mehler-Asada cycle and PTOX, the malate valve may alleviate an over-reduction of the electron transport chain and chloroplast stroma by exporting excess reducing equivalents from the chloroplast to the cytosol and mitochondria (Scheibe, 2004). However, the capacity of the malate valve is relatively restricted in C_3_ plants and therefore not sufficient to compensate for a long-term imbalance of photosynthetic electron transport and leaf assimilation (Backhausen et al., 2000; Hebbelmann et al., 2012).

Finally, changes in the photosynthetic proton circuit could increase the proton motive force across the thylakoid membrane, which in addition to inducing qE, would also slow down linear electron flux by “photosynthetic control” of plastoquinol reoxidation at the cytochrome b_6_f complex (Takizawa et al., 2007; Rott et al., 2011). An increased proton motive force across the thylakoid membrane could be either achieved by a decreased activity of chloroplast ATP synthase, reducing proton efflux from the thylakoid lumen, or the induction of cyclic electron flux, which would increase proton influx into the lumen (Cruz et al., 2005). However, in most C_3_ plants, during steady-state photosynthesis, cyclic electron flux seems to be rather low (Avenson et al., 2005; Livingston et al., 2010). High rates of cyclic flux have only been observed in the *hcef* mutants of *Arabidopsis* (Livingston et al., 2010) and in wild watermelon (*Citrullus lanatus*) in response to drought stress (Kohzuma et al., 2009; see below). ATP synthase activity is highly regulated and could be the predominant point of fine-tuning the photosynthetic proton circuit in response to short-term perturbations of the metabolic ATP and NADPH consumption (Kiirats et al., 2009). Decreased ATP synthase activity might occur as a direct consequence of a phosphate limitation (Sharkey and Vanderveer, 1989; Takizawa et al., 2008), but could also result from different post-translational modifications of the enzyme (see below). In the long term, a down-regulation of photosynthetic electron transport by an increased acidification of the thylakoid lumen has detrimental effects on the photosynthetic apparatus, especially on the oxygen-evolving complex of PSII, on plastocyanin, and PSI (Krieger and Weis, 1993; Kramer et al., 1999; Rott et al., 2011).

Therefore, while the Mehler-Asada cycle, PTOX, the malate valve, and an increased proton motive force across the thylakoid membrane may be sufficient for a short-term adjustment of photosynthetic electron transport to a decreased metabolic ATP and NADPH consumption, they are insufficient to rebalance photosynthetic ATP and NADPH production to major long-term changes in metabolic demands. Because an excessive ROS production could directly destruct the photosynthetic apparatus and initiate cell death pathways (Danon et al., 2006; Kim et al., 2012), photosynthetic complex stoichiometry adjustments are needed to adjust the capacity of the photosynthetic apparatus to long-term changes in the metabolic demand. Usually, plants indeed manage to efficiently adjust linear electron flux capacity to metabolism. Over a wide range of environmental conditions, changes in leaf assimilation capacity are paralleled by proportional changes in linear electron flux capacity (Evans, 1987; Yamori et al., 2010). Also during leaf development and senescence, large changes in leaf assimilation capacity occur. After the photosynthetic apparatus of leaves has been fully established, the photosynthetic capacity per leaf decays again. The speed of this ontogenetic loss of assimilation capacity and the underlying molecular mechanisms vary between plant species, but again, plants need to balance linear electron flux and assimilation capacity, to avoid oxidative damage (Schöttler et al.,2004).

In the following text, first, methods to quantify the major constituents of the photosynthetic apparatus will be briefly described. Then, the adjustments of the abundances of the different photosynthetic complexes to changing environmental conditions and metabolic states will be summarized. Finally, the contribution of each of the complexes to photosynthetic flux control will be discussed.

## PHOTOSYNTHETIC COMPLEX QUANTIFICATION

The *absolute* contents of photosynthetic complexes can be quantified by several biochemical and spectroscopic approaches. Additionally, activity assays can be used to approximate the contents of both photosystems and the cytochrome b_6_f complex (for example: Jenkins and Woolhouse, 1981a, b; Camp et al., 1982), and of chloroplast ATP synthase (Chow and Anderson, 1987; Chow and Hope, 1987; Evans, 1987; Chow et al., 1988). However, it has recently been shown that ATP synthase activity is strongly regulated on the post-translational level, so that up to 50% changes in enzyme content need not alter enzyme activity (Rott et al., 2011; see below). Therefore, large changes in ATP synthase activity need not be due to altered ATP synthase accumulation. *Relative* changes of complex contents can be assessed by immunoblot analyses.

Precise biochemical quantifications of PSII are based on the binding of radioactively labeled 3-(3,4-dichlorophenyl)-1,1-dimethylurea (DCMU) or atrazine to the secondary quinone acceptor (Q_B_) binding site of PSII (for example: Chow and Anderson, 1987; Chow and Hope, 1987; Evans, 1987; Chow et al., 1988, 1990; Murchie and Horton, 1998). To assess the amount of active PSII, flash-induced oxygen yields of leaf disks can be measured (Chow and Anderson, 1987; Bailey et al., 2001). Difference absorbance signals of cytochrome b_559_ (Schöttler et al., 2007a, b; Petersen et al., 2011) and the “C_550_” signal arising from Q_A_ reduction are also used to determine PSII contents (according to McCauley and Melis, 1986; method used by: Hikosaka, 1996; Kirchhoff et al., 2002; Schöttler et al., 2004).

The cytochrome b_6_f complex is mainly quantified via difference absorbance signals of cytochrome f alone (according to Whitmarsh and Ort, 1984; method applied by: Leong and Anderson, 1984; Chow and Anderson, 1987; Chow and Hope, 1987; Evans, 1987; Chow et al., 1988; Burkey and Wells, 1996; Hikosaka, 1996) or of both cytochromes f and b_6_ (Kirchhoff et al., 2002; Schöttler et al., 2004; Petersen et al., 2011).

Photosystem I quantification is mostly based on light-induced difference absorbance signals of its reaction center chlorophyll-a dimer P_700_ in the presence of methylviologen as artificial electron acceptor and sodium ascorbate as electron donor. P_700_ photooxidation is either measured at 700 nm (for example: Chow and Anderson, 1987; Chow and Hope, 1987; Evans, 1987; Chow et al., 1988, 1990; Burkey, 1993; Miersch et al., 2000; Kirchhoff et al., 2002) or in the far-red range of the spectrum at 830–870 nm wavelength (Schöttler et al., 2004, 2007a, b; Petersen et al., 2011). Also a chemical oxidation of P_700_ has been used (Leong and Anderson, 1984; Murchie and Horton, 1998; Bailey et al., 2001). Because some of these methods yield substantially different results, a direct quantitative comparison of data published by different groups is not always possible.

The quantification of other components of the photosynthetic apparatus such as plastoquinone and plastocyanin is methodologically more challenging, and has been done only in a few studies. Plastocyanin quantification is either done immunologically (Burkey, 1993, 1994; Burkey and Wells, 1996) or via difference absorbance measurements at multiple wavelengths in the far-red range between 800 and 950 nm wavelength, to separate the plastocyanin difference absorbance changes from signals arising from P_700_ absorption (Kirchhoff et al., 2004; Schöttler et al., 2004, 2007a, b; Petersen et al., 2011).

Complex contents have to be normalized either to chlorophyll content or to a leaf area basis. Because linear electron flux and leaf assimilation are usually determined on a leaf area basis, the normalization of photosynthetic complexes to leaf area allows a rapid identification of components, which closely correlate with flux rates. However, large changes in chlorophyll content per leaf area can occur due to changes in leaf morphology. Both the chloroplast number per cell, and the number of cell layers in the parenchyma can increase (see below). Therefore, the use of a chlorophyll basis may be more appropriate for the investigation of changes in the composition of the photosynthetic apparatus in the chloroplast (Walters, 2005). The use of different normalization approaches and of different techniques to quantify the components of the photosynthetic apparatus precludes a direct comparison of data produced by different groups. Therefore, in this review, mainly qualitative changes in the composition of the photosynthetic apparatus will be discussed.

## CHANGES IN PHOTOSYNTHETIC COMPLEX CONTENTS IN RESPONSE TO ALTERED ENVIRONMENTAL CONDITIONS

### LIGHT INTENSITY ACCLIMATION

In low light, photosynthesis is limited by the excitation rate of the photosynthetic reaction centers, while with increasing light intensity, the limitation shifts from charge separation to other processes of the light reactions and of the Calvin-Benson cycle. Therefore, under light-limited conditions, plants enlarge the antenna cross section per PSII reaction center, but have a relatively low electron transport and assimilation capacity. At higher light intensities, plants increase the capacity of photosynthetic electron transport (as detailed below) and of the Calvin-Benson cycle, the latter via increased accumulation of both Rubisco (Evans, 1987; Hikosaka, 1996; Yamori et al., 2010) and Rubisco activase (Yamori et al., 2010). Increased light intensities also alter leaf morphology: the number of chloroplasts in mesophyll cells, the number of mesophyll cells per leaf area, and the density of minor veins increase, enabling plants to adjust down-stream reactions such as sucrose biosynthesis and phloem loading to the increased leaf assimilation. This augments the photoassimilate export capacity from source leaves to sink tissues (Adams et al., 2007). A higher stomatal density improving CO_2_ uptake into the leaves is also observed in high-light acclimated plants.

The capacity to cope with different light intensities varies strongly between different species. Murchie and Horton (1997) analyzed the capacity of 22 British angiosperms to adjust to changes in growth light intensity and light quality. Species preferentially growing in shaded habitats show the lowest capacity to acclimate to increasing light intensity, while plants showing a low shade association of their habitat have a larger capacity to adjust their photosynthetic apparatus. A general response to increasing light intensity is an up to five-fold increase of leaf assimilation capacity. In some plant species, this is mainly due to adaptive responses of leaf morphology resulting in increased chlorophyll contents per leaf area (see above). Other plant species mainly increase their assimilation capacity via specific changes in the composition of the photosynthetic apparatus (Murchie and Horton, 1997).

Changes in the composition of the electron transport chain during the adjustment to increasing light intensities have been analyzed in a variety of species such as *Alocasia macrorrhiza* (Chow et al., 1988), *Arabidopsis thaliana* (Bailey et al., 2001), barley (*Hordeum vulgare*, Burkey, 1993), pea (*Pisum sativum*; Leong and Anderson, 1984; Chow and Anderson, 1987; Evans, 1987), soybean (*Glycine max*; Burkey and Wells, 1996), spinach (*Spinacea oleracea*; Chow and Hope, 1987), tobacco (*Nicotiana tabacum*, Petersen et al., 2011), and morning glory (*Ipomoea tricolor*; Hikosaka, 1996). All plant species displayed similar changes in the composition of their photosynthetic apparatus (summarized in **Table 1**). Only the amplitudes of the stoichiometry adjustments differed between plant species, with spinach showing relatively modest adjustments of photosynthetic complex contents (Chow and Hope, 1987), while *Arabidopsis* displayed the largest changes (Bailey et al., 2001).

**Table 1:** Changes in photosynthetic parameters during the acclimation to increased actinic light intensities.

Parameter	Low light	Increased light
Chlorophyll/leaf area	Low	High
Chlorophyll a/b	Low	High
Assimilation capacity/leaf area	Low	High
Chloroplast number/cell	Low	High
Linear electron transport/chlorophyll	Low	High
PSII/chlorophyll	Low	High
LHCII/chlorophyll	High	Low
Cytochrome b_6_f complex/chlorophyll	Low	High
Plastocyanin/chlorophyll	Low	High
PSI/chlorophyll	Unaltered	
ATP synthase/chlorophyll	Low	High

*While the general tendencies indicated above are observed in all plants, the amplitudes of stoichiometry adjustments differ considerably between species. In some species, adjustments of leaf morphology (cell number, chloroplasts per cell) are more important than alterations of photosynthetic complex stoichiometry. When light exceeds the acclimation capacity, or downstream limitations of photosynthesis occur, chlorophyll and photosynthetic complex contents may decline again at very high light intensities. For details, please refer to the text.*

On a chlorophyll basis, the contents of PSII and the cytochrome b_6_f complex increase strongly with growth light intensity, while PSI contents do not change much (Leong and Anderson, 1984; Chow and Anderson, 1987; Evans, 1987; Anderson et al., 1988; Evans, 1988; Burkey, 1993; Hikosaka, 1996; Murchie and Horton, 1998). Only at very low growth light intensities, an increased PSI accumulation can be observed (Murchie and Horton, 1998; Bailey et al., 2001). The LHCI content closely follows that of the PSI core under all growth conditions, because the four major LHCI proteins (Lhca1-4) are stably associated with PSI (Bailey et al., 2001; Ballottari et al., 2007). The up-regulation of PSII and the cytochrome b_6_f complex usually is much more pronounced on a leaf area basis, and even PSI content increases, which is due to an increased chloroplast number per cell and a higher cell number per leaf area (summarized in **Table 1**; a specific data set of light acclimation of tobacco is shown in **Table 2**). Especially the cytochrome b_6_f complex content correlates with leaf assimilation capacity during light acclimation (Leong and Anderson, 1984; Chow and Anderson, 1987; Evans, 1987, 1988; Anderson et al., 1988; reviewed by Anderson, 1992).

**Table 2:** Acclimation of the photosynthetic apparatus of tobacco (*Nicotiana tabacum*) to increasing growth light intensities.

Light intensity	30 μE m^-2^ s^-1^	300 μE m^-2^ s^-1^	1000 μE m^-2^ s^-1^
Chlorophyll (mg m^-2^)	209.2 ± 9.7	465.5 ± 60.3	421.9 ± 59.5
Chlorophyll a/b	3.14 ± 0.47	4.07 ± 0.56	4.51 ± 0.11
Assimilation (μmol CO_2_ m^-2^ s^-1^)	8.3 ± 2.4	29.7 ± 5.7	33.8 ± 3.9
PSII [mmol (mol Chl.)^-1^]	1.61 ± 0.13	2.74 ± 0.29	3.11 ± 0.25
Cytochrome b_6_f complex [mmol (mol Chl.)^-1^]	0.57 ± 0.06	1.21 ± 0.16	1.40 ± 0.17
Plastocyanin [mmol (mol Chl.)^-1^]	3.23 ± 0.24	5.84 ± 1.23	6.55 ± 1.80
PSI [mmol (mol Chl.)^-1^]	2.02 ± 0.04	2.42 ± 0.11	2.35 ± 0.06
PSII (μmol m^-2^)	0.34 ± 0.04	1.27 ± 0.19	1.30 ± 0.16
Cytochrome b_6_f complex (μmol m^-2^)	0.12 ± 0.01	0.56 ± 0.07	0.59 ± 0.08
Plastocyanin (μmol m^-2^)	0.67 ± 0.06	2.72 ± 0.54	2.75 ± 0.82
PSI (μmol m^-2^)	0.43 ± 0.03	1.13 ± 0.17	0.99 ± 0.15

*Young leaves, which had just established their maximum photosynthetic capacity, were analyzed. Plants were grown under long-day conditions (16 h light, 22°C, 75% relative humidity). Chlorophyll content per leaf area, chlorophyll a/b ratio, and CO_2_-saturated leaf assimilation capacity are shown in the top part of the table. Photosynthetic complex accumulation on a chlorophyll basis is shown in the middle section. While PSI contents per chlorophyll are largely unaffected by growth light intensity, the contents of PSII, the cytochrome b_6_f complex, and plastocyanin increase with actinic light intensity. Photosynthetic complex contents per leaf area basis are shown in the bottom part of the table. Because of the strong increase of chlorophyll content per leaf area from 30 to 300 μE m^-2^ s^-1^ light intensity, the increase of complex contents per leaf area is even more pronounced than on a chlorophyll basis. PSII was quantified from difference absorbance changes of cytochrome b_559_, the cytochrome b_6_f complex from differences absorbance changes of both cytochromes f and b_6_, and PSI from light-induced difference absorbance changes of P_700_. For detailed descriptions of the methods, please refer to Schöttler et al. (2007a), and Rott et al. (2011). The values represent averages of at least five independent plants ± standard deviation. Data for plants grown at 30 and 1000 μE m^-2^ s^-1^ are taken from Petersen et al. (2011); the data set obtained for plants grown at 300 μE m^-2^ s^-1^ light intensity is unpublished.*

Because the LHCII content decreases proportionally to the up-regulation of PSII reaction centers at increasing light intensities, usually, the total amount of chlorophyll associated with PSII-LHCII, relative to that associated with PSI and its antenna system, remains unaltered (Murchie and Horton, 1998; Bailey et al., 2001). A large antenna per PSII unit increases the excitation rate of the reaction center, which may help to minimize phototoxic side reactions in low light, because the reduction of the plastosemiquinone radical (Q_B_^-^) to the fully reduced plastoquinol is accelerated. If the semiquinone radical is not rapidly reduced, charge recombinations with the oxygen evolving complex can result in the production of singlet oxygen, which then damages PSII and causes low-light PSII photoinhibition (Keren and Krieger-Liszkay, 2011). Therefore, an increased antenna cross section under low-light conditions may reduce the rate of ROS production in PSII. On the other hand, a strong increase of the antenna cross section decreases the trapping efficiency of excitons in the PSII reaction center. While in high-light acclimated *Arabidopsis* plants, the quantum efficiency of PSII photochemistry is 91%, it decreases to 84% in low-light acclimated plants, due to the longer lifetime of the excited states in the larger PSII antenna (Wientjes et al., 2013b). Thus, the increased loss of excitation energy due to internal conversion into heat or chlorophyll-a fluorescence may restrict the maximum PSII antenna cross section (Wientjes et al., 2013b). On the other hand, a large LHCII antenna may be more nitrogen efficient, in that slightly more chlorophyll per protein can be bound by the LHCII than by the PSII reaction center proteins. Under extreme low-light conditions, the lower investment into nitrate reduction needed for LHCII production might be advantageous (Evans, 1987; Walters, 2005).

Light-intensity dependent changes in plastocyanin contents have been rarely investigated. A strong increase in plastocyanin content with the light intensity was observed in barley (*Hordeum vulgare*, Burkey, 1993) and in soybean (Burkey and Wells, 1996). Because cytochrome b_6_f complex and PSII contents increased less strongly, plastocyanin displayed the best correlation with linear electron flux capacity. The activity of chloroplast ATP synthase has been found to be highly responsive to light intensity, and usually, it is co-regulated with the cytochrome b_6_f complex (Leong and Anderson, 1984; Chow and Anderson, 1987; Evans, 1987, 1988; Anderson et al., 1988;). However, because of the strong post-translational regulation of ATP synthase activity, this need not indicate proportional changes in ATP synthase abundance (Rott et al., 2011).

Therefore, during light acclimation, the contents of PSII and the cytochrome b_6_f complex, but possibly also of ATP synthase and sometimes plastocyanin closely correlate with linear electron flux, Rubisco content and leaf assimilation capacity, while LHCII abundance is repressed by high light and PSI abundance does not change much (**Table 1**). When the light intensity exceeds the high-light acclimation capacity of plants, leading to oxidative stress, or photosynthesis becomes strongly limited by the capacity of down-stream reactions such as sucrose synthesis and photoassimilate export from the source leaves, the photosynthetic apparatus may get damaged, and the amounts of photosynthetic complexes decrease.

While large differences in light acclimation capacity between species are unsurprising, even between different genotypes of the same species, large differences in the light acclimation capacity can be observed. While the *Arabidopsis thaliana* accessions Col-0, Cape Verdi Island (Cvi), C24, and Nossen (No) show very weak or no increases in leaf assimilation capacity in response to a fourfold increase in irradiance, other *Arabidopsis* accessions such as Martuba (Mt) increase their assimilation capacity more than twofold (Athanasiou et al., 2010). Large differences in light acclimation were also observed between soybean genotypes (Burkey and Wells, 1996).

When plants are transferred from one light environment to another, their capacity to adjust to the new light regime is often strongly leaf-age dependent. Young expanding leaves show a high capacity to adjust to changes in light intensity, while mature leaves, which are fully expanded prior to the light shift, display a much lower capacity of adjustment. This is largely due to morphological constraints, because neither stomatal density nor vein density can be altered in a mature leaf, so that an up-regulation of photosynthetic capacity would result in a down-stream limitation of photosynthesis by phloem loading and photoassimilate export (Adams et al., 2007). Furthermore, photosynthetic complex biogenesis is strongly down-regulated in mature, fully expanded leaves. Because complex biogenesis cannot be rapidly reactivated, the acclimation capacity of mature leaves is highly restricted also on the chloroplast level ((Schöttler et al., 2007a; Krech et al., 2012).

### LIGHT QUALITY ACCLIMATION

While short-term exposure of plants to light preferentially exciting PSII (“PSII light”) or PSI (“PSI light”) initiates “state transitions” to rebalance excitation rates of both photosystems, a longer exposition of plants to either “PSII light” or “PSI light” results in major adjustments of photosystem stoichiometry. In pea (*Pisum sativum*), growth in “PSI light” results in a 35% increase of PSII content per chlorophyll, relative to plants grown in “PSII light,” while PSI contents are decreased by 40%. As a consequence, while in “PSII light,” the ratio of PSII to PSI is close to one, growing plants in “PSI light” results in a more than twofold higher amount of PSII than PSI. The content of the cytochrome b_6_f complex remains unaltered (Chow et al., 1990). The photosystem stoichiometry adjustments ensure that despite of major differences in light quality, the excitation rates of both photosystems are balanced, so that the quantum efficiency of CO_2_ assimilation (measured under CO_2_-saturated conditions) is close to the theoretical minimum value of about 10 quanta per assimilated CO_2_. When plants acclimated to one light quality are transferred into the opposite light quality, the quantum efficiency of CO_2_ fixation is significantly reduced (Chow et al., 1990). However, within a few days after the transfer, photosystem stoichiometry of the plants is adjusted, so that they are indistinguishable from those of plants constitutively grown under the same light regime (Kim et al., 1993). Similar changes in photosystem stoichiometry have been observed in barley (*Hordeum vulgare*; Kim et al., 1993) and in mustard (*Sinapis alba*) seedlings (Pfannschmidt et al., 1999). However, when mustard seedlings pre-acclimated to “PSII light” are transferred into “PSI light” and vice versa, the responses of photosystem stoichiometries to the light shift are dramatically more pronounced (Pfannschmidt et al., 1999). *Arabidopsis* plants transferred from one light quality to the other do not display strong changes in complex contents (Dietzel et al., 2011): PSII contents per chlorophyll remain unaltered, independent of the light quality, while PSI contents are 25% higher in “PSII light” than in “PSI light.” Remarkably, the cytochrome b_6_f complex responds most strongly: its content is very low in “PSI light,” and increases by 50% in “PSII light.”

### DROUGHT STRESS

Upon drought stress, stomatal closure is induced to reduce transpiration, and thereby CO_2_ availability to the Calvin-Benson cycle may become limiting (Flexas et al., 2004). Increased photorespiration cannot fully compensate for decreased CO_2_availability as an alternative sink for ATP and NADPH. Additionally, both photorespiration and the Calvin-Benson cycle may be repressed in response to severe drought stress by a biochemical limitation of primary metabolism, even though Rubisco levels are largely unaffected by drought (Gimenez et al., 1992; Tezara et al., 1999; Lawlor and Tezara, 2009). As a consequence, in response to long-term drought stress, photosynthetic electron transport needs to be repressed, to avoid a large imbalance between electron transport capacity and the metabolic demand. In sunflower (*Helianthus annuus*), ATP synthase content and activity are strongly decreased, so that ATP availability becomes limiting for the regeneration of Ribulose-1,5-bisphosphate, further slowing down the activity of the Calvin-Benson cycle (Tezara et al., 1999).

Also wild watermelon (*Citrullus lanatus*) strongly down-regulates linear electron flux in response to long-term drought stress. ATP synthase contents are reduced by about 50%, but the cytochrome b_6_f complex content is even more strongly decreased, down to only 25% of that of unstressed control plants. The contents of both photosystems remain largely unaltered during drought stress (Kohzuma et al., 2009). ATP synthase activity decreases even more strongly than ATP synthase content, so that ultimately, ATP synthase activity becomes rate-limiting for the light reactions. Due to the decrease of ATP synthase activity and proton efflux from the lumen, and possibly the induction of cyclic electron flux, the steady-state proton motive force across the thylakoid membrane is strongly increased. The strong acidification of the thylakoid lumen further slows down linear electron flux via “photosynthetic control” and allows the onset of strong photoprotective qE (Kohzuma et al., 2009).

### COLD STRESS

Evergreen conifers retain most of their chlorophyll throughout the winter, which facilitates the rapid recovery of photosynthesis when temperature increases again in spring. Especially at subzero temperature, light capture and photosynthetic electron transport have to be balanced to a strongly decreased metabolic activity, in order to prevent oxidative destruction of the photosynthetic apparatus. Therefore, during winter acclimation, photosynthesis is gradually down-regulated and ultimately, linear electron flux is almost completely repressed (Sveshnikov et al., 2006; Tanaka, 2007). The amounts of the major photosynthetic complexes decrease during acclimation: in Jack pine (*Pinus banksiana*), the amount of D1 protein is reduced by 60 to 80%, while PSI and LHCI are slightly less affected (Busch et al., 2007, 2008). In the Subalpine Fir (*Abies lasiocarpa*), the D1 protein content decreases to a similar extent as in Jack pine, and the PsbO protein is almost completely absent (Zarter et al., 2006). In *P. banksiana* the amount of the cytochrome b_6_f complex is repressed by about 60%, but plastocyanin and ATP synthase contents are only marginally affected (Busch et al., 2008; Savitch et al., 2010). While the amounts of most LHCII decrease considerably (Busch et al., 2007, 2008; Verhoeven et al., 2009; Savitch et al., 2010), the relative abundances of PSII-associated Lhcb1, Lhcb3, and Lhcb6 increase about two-fold in *P. banksiana* and *P. contorta* (Busch et al., 2008; Savitch et al., 2010).

Overall, these changes in complex stoichiometries ensure a very low level of linear electron transport that can limit oxidative damage during winter periods. Still, maintaining chlorophyll content (i.e., light-harvesting capacity) means that almost all light absorbed by the plant is in excess and has to be dissipated harmlessly. Thus, down-regulation of linear electron transport is accompanied by the induction of several photoprotective mechanisms and energy dissipation pathways: the PsbS protein, the photoprotective ELIP-like proteins, and the amounts of protective pigments, such as lutein and carotenoids are up-regulated relative to chlorophyll (Matsubara et al., 2003; Zarter et al., 2006; Savitch et al., 2010; Collakova et al., 2013). Because also high levels of xanthophyll cycle pigments and lower epoxidation levels are sustained, a non-regulated, continuously high level of NPQ has been suggested to be a main photoprotective mechanism during winter in pine species (Öquist and Huner, 2003; Ivanov et al., 2006; Sveshnikov et al., 2006; Savitch et al., 2010). Additionally, both the Mehler reaction (Öquist and Huner, 2003; Savitch et al., 2010) and PTOX are induced (Busch et al., 2008; Savitch et al., 2010). The ROS detoxifying functions are also important upon cold acclimation, as shown by the upregulation of several genes encoding enzymes of the ascorbate-gluthatione cycle and the ascorbate biosynthesis pathway (Collakova et al., 2013).

## CHANGES IN PHOTOSYNTHETIC COMPLEX CONTENTS DURING LEAF ONTOGENESIS

Different plant species show vastly different changes in assimilation capacity and in the composition of their photosynthetic apparatus during leaf ontogenesis. During early phases of leaf development, the photosynthetic apparatus is established and the leaf develops from a sink tissue dependent on net import of photoassimilates into a source leaf exporting assimilates (Meng et al., 2001). Once full leaf expansion is reached, large differences exist in the ontogenetic programs of higher plants, especially between cereal crops and agriculturally relevant dicots.

### CEREAL CROPS

In wheat (*Triticum aestivum*), barley (*Hordeum vulgare*), and rice (*Oryza sativa*), chlorophyll content, Rubisco content, and leaf assimilation capacity start to decline soon after the leaf is fully expanded (Camp et al., 1982; Kura-Hotta et al., 1990; Ono et al., 1995; Miersch et al., 2000; Tang et al., 2005). However, this is not due to a remodeling of the photosynthetic apparatus in the chloroplasts. Instead, chloroplasts seem to be degraded *in toto*. Also, because chloroplasts shrink significantly, the mesophyll cell volume occupied by chloroplasts decreases strongly (Peoples et al., 1980; Camp et al., 1982; Wittenbach et al., 1982; Kura-Hotta et al., 1990; Ono et al., 1995). Because up to 75% of total reduced nitrogen in cereal leaves is found in the photosynthetic machinery, the complete degradation of chloroplasts in mature leaves may increase the nitrogen use efficiency of the plant. Cereals have been selected for an efficient nutrient remobilization from older to younger leaves and especially to the grains during hundreds of years of breeding (Gregersen et al., 2008). The precise mechanism underlying the decreased size and the *in toto* degradation of chloroplasts in cereals is still unknown: degradation of thylakoid membranes and the release of “Rubisco-containing bodies,” vesicles enriched in stromal proteins during the process of autophagy, but also the phagocytosis of entire chloroplasts by the vacuole have been observed (Hörtensteiner and Feller, 2002; Gregersen et al., 2008; Wada et al., 2009). The composition of the photosynthetic apparatus of the remaining chloroplasts remains unaltered in most cereal crops until the late phases of senescence, when also photosynthetic complex content per chlorophyll starts to decrease. Interestingly, in most cereal crops, the loss of PSI and cytochrome b_6_f complex precedes the loss of PSII. LHCII remains stable until the last stages of senescence (Camp et al., 1982; Holloway et al., 1983; Hidema et al., 1991, 1992; Humbeck et al., 1996; Miersch et al., 2000; Tang et al., 2005). For chloroplast ATP synthase, both a parallel decline with the cytochrome b_6_f complex (Hidema et al., 1991) and a high stability until the final phase of leaf senescence (Camp et al., 1982) have been reported.

### DICOTYLEDONS

Similar to cereal crops, the dicot common bean (*Phaseolus vulgaris*) reaches maximum leaf assimilation capacity shortly after its leaves are fully expanded, and during the next four weeks, displays a continuous leaf-age dependent loss of chlorophyll content down to 25% of that of young leaves. However, the underlying mechanisms are completely different from those in cereal crops, in that the number of chloroplasts per mesophyll cell remains unaltered until the final phase of leaf senescence. In parallel to chlorophyll content, linear electron flux *per chlorophyll* decreases down to 20% of the capacity of young leaves, so that electron transport *per leaf area* goes down to less than 5% of the capacity of young leaves (Jenkins and Woolhouse, 1981a). Because the activity of the two photosystems, expressed on a chlorophyll basis, remains largely unaltered, the strong repression of linear electron flux is likely attributable to a repression of either cytochrome b_6_f complex or one of the mobile redox carriers, plastoquinone and plastocyanin (Jenkins and Woolhouse, 1981b). Roberts et al. (1987) and Prakash et al. (2001) confirmed a selective depletion of the cytochrome b_6_f complex. Different to cereal crops, PSII contents decreased more rapidly than those of PSI and chloroplast ATP synthase (Prakash et al., 2001). During developmental senescence in morning glory (*Ipomoea tricolor*), besides of strong decreases in Rubisco and cytochrome b_6_f complex content, also a strong reduction of PSII content was observed, while PSI content again remained unaltered when senescing leaves were shaded by the younger leaves. When shading was abolished by vertical growth of the plants, the developmental decrease in photosynthetic capacity, Rubisco content, and of the different constituents of the photosynthetic apparatus was delayed, indicating that shading may be a major determinant of the progression of leaf senescence (Hikosaka, 1996).

Also tobacco shows a continuous decline of leaf assimilation capacity after the source leaves are fully developed. Assimilation capacity per leaf area decreases to less than 10% of the maximum capacity found in young leaves prior to significant reductions of leaf chlorophyll content (Schöttler et al., 2004). Photosynthetic electron transport is repressed in parallel with leaf assimilation capacity by a proportional down-regulation of the cytochrome b_6_f complex, plastocyanin, and chloroplast ATP synthase. The contents of both photosystems, their antenna proteins, and the size of the plastoquinone pool remain largely unaltered during leaf ontogenesis (Schöttler et al., 2004, 2007a). Therefore, despite the massive decrease in leaf assimilation capacity, a large fraction of leaf nitrogen is only remobilized during the final phase of developmental leaf senescence in tobacco.

The down-regulation of leaf assimilation and photosynthetic electron transport during leaf senescence usually is a slow process proceeding over several weeks. A much more rapid repression of the Calvin-Benson cycle and of photosynthetic electron transport can occur in response to a sudden sink limitation of photosynthesis, due to nutrient limitations restricting plant growth, to the removal of sink organs from the plants, or to restrictions in the phloem-mediated photoassimilate export from source leaves to sink tissues. The resulting accumulation of photoassimilates in source leaves is perceived via “sugar sensing” and represses the Calvin-Benson cycle and photosynthetic electron transport (Koch, 1996; Pego et al., 2000). When phloem loading is blocked in tobacco mutants expressing an apoplastic invertase, leaf assimilation and linear electron flux are rapidly repressed (Schöttler et al., 2004). However, the co-regulation of cytochrome b_6_f complex and plastocyanin contents observed during ontogenetic leaf senescence is abolished. Rather, photosynthetic electron transport is limited by a rapid repression of plastocyanin contents, while the decrease of the cytochrome b_6_f complex is delayed (Schöttler et al., 2004).

## PHOTOSYNTHETIC FLUX CONTROL

In metabolic control analysis, the contribution of the different enzymes of a metabolic pathway to the control of total flux through that pathway is analyzed. To this end, the activity of each enzyme of the pathway is step-wise repressed via the application of increasing concentrations of specific inhibitors. If no specific inhibitor is available, enzyme contents can be decreased by genetic approaches, and then, the effects of decreased enzyme abundance on its activity and the total flux through the pathway can be compared. A flux control coefficient close to zero means that an enzyme does not exert any control over the flux, it is non-limiting. A control coefficient of one indicates that an enzyme exerts total control, so that any change of its activity will result in a proportional change of flux through the entire pathway (Fridlyand and Scheibe, 2000). During the last decades, the contribution of most enzymes of the Calvin cycle to the control of leaf assimilation has been studied in detail (reviewed by Fridlyand and Scheibe, 2000; Raines, 2003). Also the contribution of several components of the electron transport chain to photosynthetic flux control has been elucidated. Surprisingly, high flux control coefficients have been measured for several components of the electron transport chain. Systematic correlations of the contents of the different constituents of the photosynthetic apparatus with either linear electron flux capacity alone or total leaf assimilation capacity can be used to determine which component exerts control under which environmental condition and developmental state of the leaf.

### PSII

The very minor adjustments of PSII contents in response to conditions necessitating a down-regulation of linear electron flux, for example during leaf senescence (Jenkins and Woolhouse, 1981b; Holloway et al., 1983; Roberts et al., 1987; Schöttler et al., 2004) and drought stress (Kohzuma et al., 2009), strongly suggest that PSII does not contribute to photosynthetic flux control. The pronounced increase in PSII contents with increasing light intensities occurring on expense of the LHCII abundance (see Light Intensity Acclimation) also need not indicate a limiting function of PSII under these conditions. Instead, it enables plants to optimize exciton trapping efficiency per PSII (see above). An exception is the down-regulation of linear electron flux during cold stress in evergreen conifers, where PSII contents are strongly repressed, relative to the other photosynthetic complexes (Zarter et al., 2006; Busch et al., 2007, 2008). However, at subzero temperatures, inhibited diffusion of the mobile redox carriers plastoquinone and plastocyanin might limit linear electron flux.

The hypothesis that PSII does not contribute to photosynthetic flux control is well in line with the fact that all reactions catalyzed by PSII are substantially faster than plastoquinol re-oxidation at the cytochrome b_6_f complex, and that PSII is always present in excess amounts, relative to the cytochrome b_6_f complex. Accordingly, in eukaryotic algae, photoinhibition of up to 50% of all PSII does not have a major influence on linear electron flux capacity, but only decreases the quantum efficiency of photosynthesis (Kana et al., 2002). Antisense repression of the PsbO subunit of the oxygen evolving complex of PSII to less than 50% of wild-type amounts in *Arabidopsis* does not alter photosynthetic capacity. Again, only the quantum efficiency of CO_2_ fixation under limiting light is reduced (Dwyer et al., 2012).

However, the step-wise inhibition of PSII activity by DCMU application results in a proportional decrease of linear electron flux in higher plants, indicating a flux control coefficient of PSII close to one (Kirchhoff et al., 2000). This observation can be explained by a massive restriction of plastoquinone diffusion within the thylakoid membrane. Because of a high thylakoid membrane area occupation by proteins, which exceeds 75% of membrane area in the grana, the lipid phase available for plastoquinone diffusion is highly restricted, especially because most of the remaining lipids are stably associated with the photosynthetic complexes as boundary lipids (Kirchhoff et al., 2002). Therefore, most of the thylakoid membrane space in the granum is filled with diffusion obstacles, especially PSII and LHCII, so that plastoquinone cannot diffuse freely over long distances, but is trapped within diffusion microdomains. It can only rapidly connect PSII in the grana with cytochrome b_6_f complexes in their close vicinity (Kirchhoff et al., 2000; Tremmel et al., 2003). Consequently, when one PSII is inactivated by the addition of DCMU, the nearest cytochrome b_6_f complex is inactivated as well, because it is disconnected from its electron donor. This also explains the discrepancy between plants with antisense repression of PSII and plants treated with DCMU: antisense repression decreases total PSII content, but the thylakoid structure can be adjusted to this, so that all cytochrome b_6_f complexes can still be efficiently connected to PSII units.

### CYTOCHROME B_6_F COMPLEX

Most studies on changes in electron transport and leaf assimilation capacity revealed a close correlation between electron transport capacity and the content of the cytochrome b_6_f complex (see above). Because the cytochrome b_6_f complex catalyzes the slowest reaction of linear electron flux, plastoquinol reoxidation, and usually is present in sub-stoichiometric or at best equimolar amounts relative to the two photosystems, it has been suggested to be the major site of photosynthetic flux control in higher plants (Haehnel, 1984; Anderson, 1992; Hope, 2000). A major role of the cytochrome b_6_f complex in flux control was ultimately confirmed via the specific repression of its activity by the use of inhibitors (Kirchhoff et al., 2000), and by the genetic repression of its accumulation in *PetC*-antisense plants (Price et al., 1995, 1998; Anderson et al., 1997; Yamori et al., 2011). Both inhibitor treatments and antisense repression of the Rieske protein of the cytochrome b_6_f complex result in proportional decreases of linear electron flux and of leaf assimilation, establishing a flux control coefficient close to one for the cytochrome b_6_f complex (Price et al., 1995; Kirchhoff et al., 2000).

### PLASTOCYANIN

The contribution of plastocyanin to photosynthetic flux control is still matter of debate. During light acclimation in barley, Burkey (1993) observed a much better correlation between plastocyanin content than between any other complex content and electron transport capacity. Also during the rapid repression of leaf assimilation in response to a sink limitation of photosynthesis, Schöttler et al., (2004) identified plastocyanin as the rate-limiting component of the electron transport chain. A limiting function of plastocyanin in linear electron flux is also supported by studies in different barley and soybean genotypes: plastocyanin showed by far the best correlation of any component of the electron transport chain with linear electron flux capacity (Burkey, 1994; Burkey et al., 1996). However, in *Arabidopsis thaliana*, a 60 to 80% repression of plastocyanin content does not result in any photosynthetic defect and growth phenotype, and even a 90% repression of plastocyanin content has only minor effects on linear electron flux and plant growth, clearly showing that in *Arabidopsis*, plastocyanin does not limit linear electron flux (Pesaresi et al., 2009). However, *Arabidopsis* is unusual in that, different to most other plant species investigated, it accumulates large amounts of plastocyanin, irrespective of growth light intensity, light quality, and the developmental state of the leaf, so that a large excess of plastocyanin may be present (Mark A. Schöttler, unpublished results). In *Arabidopsis*, plastocyanin may function as a major copper storage protein (Puig et al., 2007). Therefore, a role of plastocyanin in photosynthetic flux control in other plant species cannot be excluded.

Plastocyanin has to connect the cytochrome b_6_f complexes in the grana with PSI in the grana margins, end membranes, and stroma lamellae. However, plastocyanin diffusion in the thylakoid lumen is restricted (Kirchhoff et al., 2004; Schöttler et al., 2004, 2007b). Because the diameter of plastocyanin is similar to the diameter of the thylakoid lumen in darkness and at low-light conditions, the lumen can be considered as a two-dimensional diffusion space. The oxygen-evolving complexes of PSII protruding from both sites into the lumen form diffusion obstacles for plastocyanin (Hope, 2000). When the lumen diameter is decreased by hyperosmotic conditions, plastocyanin diffusion in the thylakoid lumen is almost completely abolished (Cruz et al., 2001). At higher light intensities, the efficiency of plastocyanin diffusion may increase, because high light induces swelling of the thylakoid lumen, so that the lumen width is almost doubled, and the relative luminal space occupied by the oxygen evolving complexes is significantly reduced (Kirchhoff et al., 2011).

### PSI

With the exception of the early degradation of PSI during leaf senescence in cereal crops, no indications exist for an adjustment of PSI contents to changing environmental conditions. Also on a theoretical basis, it appears unlikely that PSI plays a limiting role in photosynthetic electron transport. Similar to PSII, it is usually present in excess amounts relative to the cytochrome b_6_f complex, and electron transfer from plastocyanin to ferredoxin is much faster than the redox reactions catalyzed by the cytochrome b_6_f complex (Haehnel, 1984; Hope, 2000). However, a precise analysis of the role of PSI in photosynthetic flux control has not been possible so far, because no specific inhibitors of PSI exist to down-regulate its activity and determine the impact on photosynthesis. Also the genetic repression of PSI contents is more difficult than that of PSII or the cytochrome b_6_f complex, because the essential reaction center subunits binding the redox-active cofactors are encoded in the chloroplast genome. Therefore, so far, no detailed flux control analysis has been possible. However, circumstantial evidence supports the conclusion that PSI is not involved in photosynthetic flux control: a diminished accumulation of the essential auxiliary protein Ycf3, which is required for the assembly of the stromal ridge of PSI involved in ferredoxin binding, results in a 25% reduction of PSI contents under high-light conditions in tobacco, but the accumulation of the other photosynthetic complexes, linear electron flux capacity, and plant growth are unaltered, relative to the wild type (Petersen et al., 2011). Even an 80% reduction in PSI content due to the antisense repression of the essential nuclear-encoded assembly factor Y3IP1, which interacts with Ycf3 in the assembly of the stromal ridge, has no effect on the accumulation of the other photosynthetic complexes and only moderately delays plant growth (Albus et al., 2010). An even more severe repression of PSI contents down to less than 10% of wild-type amounts due to an attenuated translation of the PsaA reaction center subunit still allows the transformants to grow autotrophically, even though growth is massively retarded (Krech et al., 2012).

### FERREDOXIN AND FERREDOXIN NADP+ OXIDOREDUCTASE

In leaves of higher plants, usually two different isoforms of ferredoxin can function in electron transport (Hanke et al., 2004). The repression of the major ferredoxin isoform, Fd2, in *Arabidopsis* strongly impairs linear electron transport, indicating that ferredoxin may play a limiting role in photosynthetic electron transport (Hanke and Hase, 2008). Already minor reductions of the NADP^+^-dependent ferredoxin oxidoreductase (FNR) by in antisense repression severely affect electron transport, leaf assimilation, and growth (Hajirezaei et al., 2002). However, because so far, the accumulation of neither ferredoxin nor FNR during the adjustment of the photosynthetic apparatus to changing environmental conditions and during leaf ontogenesis has been systematically assessed, it is impossible to predict a possible limiting function of them during the acclimation of the photosynthetic apparatus.

### CHLOROPLAST ATP SYNTHASE

ATP synthase content increases in parallel with the cytochrome b_6_f complex when growth light intensity is increased (Leong and Anderson, 1984; Chow and Anderson, 1987; Chow and Hope, 1987; Evans, 1987, 1988; Anderson et al., 1988). In response to a reduced metabolic demand for ATP and NADPH during drought stress in wild watermelon (Kohzuma et al., 2009) and during leaf senescence in dicots, both complexes are repressed in parallel (Schöttler et al., 2007a). This strict co-regulation of ATP synthase and cytochrome b_6_f complex suggests that ATP synthase may have a role in photosynthetic flux control. However, when ATP synthase accumulation is repressed via antisense approaches against the nuclear-encoded γ-subunit (AtpC) and by decreased translation of the plastome-encoded β-subunit (AtpB), an up to 50% reduction in ATP synthase content does not alter ATP synthase activity, linear electron flux, and leaf assimilation. Obviously, a large fraction of chloroplast ATP synthase is inactive in wild-type tobacco (Rott et al., 2011). This could be due to post-translational modifications, especially the reversible phosphorylation of the β-subunit and the interaction of the phosphorylated enzyme with 14-3-3 proteins (Bunney et al., 2001; del Riego et al., 2006; Reiland et al., 2009).

Only when ATP synthase contents decrease to less than 50% of wild-type amounts, ATP synthase activity and assimilation decrease linearly with ATP synthase content, because then, all remaining ATP synthase is in its active form (Rott et al., 2011). Similar results were obtained also for tobacco antisense transformants targeted against the nuclear-encoded δ-subunit (AtpD; Yamori et al., 2011). Decreased leaf assimilation in ATP synthase mutants is not exclusively attributable to restricted ATP synthesis. Because ATP synthase limits the photosynthetic proton circuit, an increased acidification of the thylakoid lumen inhibits linear electron flux via photosynthetic control of plastoquinol reoxidation. Also, qE is already induced at low light intensities, so that the quantum efficiency of CO_2_fixation is severely reduced (Rott et al., 2011). Such a situation is also observed in wild watermelon under severe drought stress (Kohzuma et al., 2009).

These data suggest that ATP synthase activity and linear electron flux, either controlled by the cytochrome b_6_f complex or plastocyanin contents, need to be co-regulated to balance the photosynthetic proton circuit so that under non-stressed conditions, lumen acidification is sufficient to drive ATP synthesis, but does not activate qE and photosynthetic control of linear electron flux. However, when ATP synthase activity slows down due to slow ATP consumption by the Calvin-Benson cycle and a limitation by phosphate availability, the thylakoid lumen becomes sufficiently acidic to slow down linear electron transport via photosynthetic control and trigger photoprotective qE.

In summary, even though the cytochrome b_6_f complex seems to be the predominant point of photosynthetic flux control that limits both linear electron flux and leaf assimilation, under specific metabolic conditions such as a severe sink limitation of photosynthesis or drought stress, other components of the photosynthetic apparatus such as plastocyanin and ATP synthase may play a role in photosynthetic flux control as well.

## SUMMARY AND OUTLOOK

More than 30 years of research have resulted in detailed knowledge of the adjustment of the major photosynthetic complexes to changes in the light environment and in the metabolic demand for ATP and NADPH during leaf ontogenesis. Differences in light acclimation capacity of different species can be attributed to an evolutionary adaptation to different levels of shade association. The dramatic differences in leaf ontogenesis and senescence especially observed between agricultural plant species can be explained by the selection for plants with either a high nitrogen use efficiency, such as several cereals, or for plants with high yields of leaf biomass (for example tobacco).

The vast majority of acclimation studies have been performed in the 1980s and 1990s, when several important regulatory components involved in the fine-tuning of photosynthesis were still unknown: therefore, our knowledge of adaptive changes in the accumulation of various components involved in cyclic electron flux such as the NDH complex and the recently identified PGR5 and PGRL1 proteins during leaf ontogenesis and environmental acclimation is still limited (Hertle et al., 2013; Leister and Shikanai, 2013). Also the responses of important regulators of state transitions and factors controlling thylakoid topology such as the STN7 and STN8 kinases and the CURT1 proteins (Armbruster et al., 2013) to environmental perturbations and during leaf ontogenesis are mostly unknown. The components on the PSI acceptor side, ferredoxin and FNR, and possible changes in chloroplast lipid composition have been addressed only in a few acclimation studies. Finally, our knowledge of the mechanisms underlying the stoichiometry adjustments is still limited. While detailed information exists on signals initiating acclimation responses (recently reviewed by: Pogson et al., 2008; Kleine et al., 2009; Foyer et al., 2012; Pfannschmidt and Yang, 2012), much less is known about limiting steps, which ultimately determine complex accumulation in higher plants. Indications exist for regulation of photosynthetic complex biogenesis on the transcriptional level, on the level of mRNA maturation (especially in case of the chloroplast-encoded subunits), of mRNA translation, and on the level of complex assembly, which requires multiple auxiliary proteins (recently reviewed by: Adam et al., 2011; Schöttler et al., 2011; Lyska et al., 2013; Nickelsen and Rengstl, 2013). Complex stability and degradation may be highly regulated as well.

We believe that two major future directions of research could be taken to fully understand photosynthetic stoichiometry adjustments:

(1) “Systems” approaches should enable us to dissect the acclimation process of the entire photosynthetic apparatus to single parameter changes and determine the signals and mechanisms controlling acclimation responses. Due to the excellent knowledge of light acclimation responses of the photosynthetic complexes, first experiments could be based on changes in growth light intensity and light quality. Changes in photosynthetic gene expression, complex biogenesis and photosynthetic activity could be determined with high temporal resolution after the light shift, and the composition of the entire photosynthetic apparatus, including lowly abundant regulatory proteins, could be determined using a combination of biophysical and proteomics approaches. Such single parameter experiments could be expanded to environmental perturbations, which so far have not been in the center of interest: for example, to our knowledge, not a single analysis of photosynthetic complex stoichiometry adjustments in response to heat stress has been published. A combination of such single parameter studies should allow the identification of highly regulated factors, which control the biogenesis and accumulation of the different components of the photosynthetic apparatus.

(2) Most acclimation studies of the photosynthetic apparatus have been performed under highly controlled growth conditions in the laboratory. Many mutants affected in components of the photosynthetic apparatus do not show obvious photosynthetic defects under these controlled conditions. However, phenotypes occur or become more pronounced when mutants are exposed to fluctuating environmental conditions. For example, growth of different *Lhca* and *Lhcb* mutants (Ganeteg et al., 2004) and of mutants defective in NPQ (Külheim and Jansson, 2005) either in the field or under fluctuating conditions in the laboratory resulted in clear photosynthetic phenotypes and effects on plant fitness. Similarly, the use of rapidly changing growth light intensities simulating sun flecks resulted in drastic photosynthetic defects in the *stn7* and the *pgr5* mutant (Tikkanen et al., 2010; Grieco et al., 2012; Suorsa et al., 2012). Therefore, one future focus of acclimation studies could be the systematic investigation of responses of the entire photosynthetic apparatus to periodic changes of environmental parameters with the final goal of understanding how plants as sessile organisms are capable of adapting to large environmental changes.

## Conflict of Interest Statement

The authors declare that the research was conducted in the absence of any commercial or financial relationships that could be construed as a potential conflict of interest.
